# Ofatumumab for the Treatment of Anti‐Neurofascin 155 Autoimmune Nodopathy: A Case Series

**DOI:** 10.1002/brb3.70717

**Published:** 2025-08-04

**Authors:** Kalam Choi, Peicai Fu, Jinyi Yan, Zhijun Li

**Affiliations:** ^1^ Department of Neurology, Tongji Hospital, Tongji Medical College Huazhong University of Science and Technology Wuhan China

**Keywords:** anti‐neurofascin 155, autoimmune nodopathy, chronic inflammatory, demyelinating polyneuropathy, Ofatumumab

## Abstract

**Introduction:**

Autoimmune nodopathy (AN) is a rare immune‐mediated peripheral neuropathy, the diagnosis and treatment of which remain challenging. Anti‐neurofascin‐155 (NF155) AN present with weakness, tremor, ataxia, and cranial nerve involvement. Ofatumumab, a second‐generation anti‐CD20 monoclonal antibody, functions by depleting B lymphocytes. This study aimed to investigate the efficacy of ofatumumab treatment in NF155 AN.

**Methods:**

We reviewed data on ofatumumab treatment of patients with anti‐NF155 AN at the Department of Neurology, Tongji Hospital, Tongji Medical College, Huazhong University of Science and Technology from January 2020 to September 2024.

**Results:**

A total of four patients with anti‐NF155 AN were included, consisting of three males and one female. The onset age ranged from 9 to 27 years. All cases exhibited distal sensory‐motor neuropathy accompanied by tremors and ataxia. All the patients received corticosteroids or other immunotherapy or both before treatment with ofatumumab. The duration of treatment with ofatumumab ranged from 6 to 25 months, with a follow‐up period of 6–28 months. The clinical improvements of patients were assessed using the Inflammatory Neuropathy Cause and Treatment Disability Score, the Medical Research Council Muscle Strength Score, and the Modified Rankin Scale. All four patients demonstrated significant clinical improvement following ofatumumab treatment, which was concurrently accompanied by varying levels of improvement in imaging and nerve conduction studies. Throughout the treatment period, the patients did not report any adverse reactions to ofatumumab.

**Conclusion:**

Our research findings suggested that ofatumumab holds significant potential for treating anti‐NF155 AN.

## Graphical abstract

Summarize the key messages of this paper Ofatumumab treatment led to significant clinical, imaging, and electrophysiological improvements in four patients with anti‐NF155 AN.1. No adverse reactions to ofatumumab were observed in any of the patients. Ofatumumab has great potential for treating anti‐NF155 AN.3.

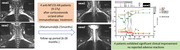

## 1 Introduction

Autoimmune nodopathy (AN) is a rare immune‐mediated peripheral neuropathy that is distinguished from chronic inflammatory demyelinating polyneuropathy (CIDP) by its unique clinical features and the absence of macrophage‐mediated inflammation or demyelination (van den Bergh et al. 2021). AN is primarily associated with immunoglobulin G4 (IgG4) antibody isotypes targeting the peripheral nodo‐paranodal region of Ranvier, such as anti‐neurofascin‐155 (NF155), anti‐NF140/186, anti‐contactin‐1 (CNTN1), and anti‐contactin‐associated protein (CASPR1), and it presents as weakness, tremor, ataxia, and cranial nerve involvement (Gupta et al. 2023).

Given the debilitating nature of immune‐mediated neuropathies, it is crucial to initiate the most effective therapy as early as possible. Corticosteroids remain among the most frequently used agents in the treatment of Autoimmune nodopathy (Martin‐Aguilar et al. 2022), and plasma exchange (PE) may further improve patient outcomes (Broers et al. 2023). However, a significant proportion of AN cases show limited response to initial intravenous immunoglobulin (IVIG) therapy, likely due to the predominance of the IgG4 antibody isotype. RTX, a chimeric anti‐CD20 antibody, has demonstrated effectiveness in treating anti‐NF155 and anti‐NF186 AN in various case studies, with reported response rates of up to 80% (Broers et al. 2023; Liu, Hu, et al. 2023; Liu, Zhou, et al. 2023).

Ofatumumab is recognized as a second‐generation anti‐CD20 monoclonal antibody that targets a distinct site on CD20 cells compared with rituximab and functions by depleting B lymphocytes. Subcutaneous administration of ofatumumab has been approved for treating patients with relapsing‐remitting multiple sclerosis (Kang and Blair 2022). Encouraging outcomes have been noted with ofatumumab in patients with various immune diseases, including AE, MN, and SLE (Zhou et al. 2023; Kaegi et al. 2021).

However, further treatment options are currently being explored for AN. A recent study found that combining the traditional immunosuppressant tacrolimus with steroids effectively managed AN cases (Yang et al. 2022). Monoclonal antibodies targeting various stages of the pathogenic mechanism of B cells, such as daratumumab and telitacicept, have also shown success in refractory AN case (Scheibe et al. 2022; Ren et al. 2023). Additionally, autologous hematopoietic stem cell transplantation has yielded promising results in a refractory AN case (Afanasiev et al. 2024).

In this study, we present a case series of patients with anti‐NF155 AN, whose condition was significantly improved following ofatumumab treatment. This study is a retrospective observational study. Patients received medication based on their medical conditions and following thorough discussions with professional physicians, without any interference in their medication decision‐making processes.

## 2 Methods

### 2.1 Patients

In this retrospective analysis, we reviewed data on ofatumumab treatment of patients with anti‐NF155 AN at the Department of Neurology, Tongji Hospital, Tongji Medical College, Huazhong University of Science and Technology from January 2020 to September 2024. A total of four patients with anti‐NF155 AN were included. The demographics, clinical characteristics, nerve conduction studies (NCS), and treatment details of the patients were collected through electronic medical records.

The diagnosis of anti‐NF155 AN was confirmed through typical clinical symptoms, electrophysiological tests, and positive serological examination, following the diagnostic criteria established in the European Academy of Neurology/Peripheral Nerve Society guidelines (van den Bergh et al. 2021).

This study was approved by the Medical Ethics Committee of Tongji College, Huazhong University of Science and Technology ([2023]‐S171), in accordance with the ethical standards of the Declaration of Helsinki. Informed consent was obtained from all patients or their legal representatives.

### 2.2 Treatment Protocol and Outcome Evaluation

Cases 1 and 2 received the individualized injection, which was resumed when the CD19 B‐cell count exceeded 10/µL. Lymphocyte subset testing was conducted approximately every 30–45 days during the initial treatment phase. After establishing a general pattern for the rebound time of CD19 B cells, the time interval between the patient's two injections could be recommended. The frequency of subsequent tests was aligned with the patient's follow‐up schedule. The conventional protocol was followed for cases 3 and 4. According to the protocol in relapsing multiple sclerosis treatment, an injection was administered on days 1, 7, and 14, followed by routine monthly doses thereafter (20 mg, IH, Q4W) (Kang and Blair 2022).

**FIGURE 1:**
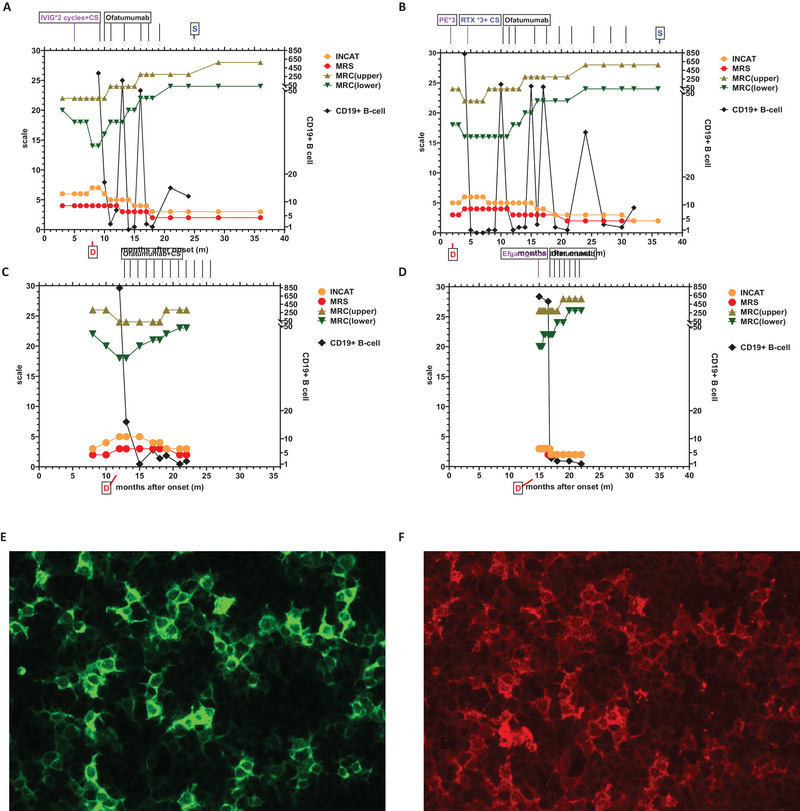
Clinical timeline and symptom changes (INCAT, MRS, MRC [upper], MRC [lower]) and CD19^+^ B cell changes in cases 1–4 (A–D). (E) Immunofluorescence showed cells labeled with e‐GFP and stably expressing NF155. (F) IgG combined with NF155 was fluorescently labeled: D indicates the timepoint of confirmed diagnosis, and S indicates the timepoint of discontinue ofatumumab. CS, corticosteroid; INCAT, Inflammatory Neuropathy Cause and Treatment Disability Score; IVIG, Intravenous Immune Globulin; MRC, Medical Research Council Muscle Strength Score; MRS, the Modified Rankin Scale; PE, plasma exchange; RTX, rituximab.

In this study, the clinical improvements of patients were assessed using the Inflammatory Neuropathy Cause and Treatment Disability Score, the Medical Research Council Muscle Strength Score, and the Modified Rankin Scale. NCS were conducted using standard electromyography techniques, including motor and sensory NCS of peripheral nerves: the median and ulnar nerves in the upper limbs, and the peroneal, tibial, and sural nerves in the lower limbs. The measured parameters included motor nerve conduction velocity (MCV), distal motor latency (DML), compound muscle action potential (CMAP) amplitude, as well as sensory nerve conduction velocity (SCV), and sensory nerve action potential (SNAP) amplitudes. All adverse events during the treatment phase were systematically recorded, including infectious, noninfectious, and hypersensitivity reactions. NF155 antibodies were identified using cell‐based indirect immunofluorescence assays (Figure 1E,F). Complete blood cell and CD19 counts were monitored.

## 3 Results

### 3.1 Case Series

#### 3.1.1 Case 1

A 10‐year‐old boy was admitted to our department in May 2022 due to “progressive limb weakness and numbness” that had persisted for over 8 months. In August 2021, the family noticed that the patient began experiencing weakness in the distal upper limbs, tremors in both hands, and difficulties with fine motor skills, such as holding chopsticks and buttoning clothes. Two days after receiving a COVID‐19 vaccine in November 2021, the patient developed weakness in both calves with foot drop, difficulty walking, and limited ability to climb stairs, accompanied by symptoms such as frequent urination. The patient visited external hospitals thrice, where NCS indicated prolonged DML and reduced MCV, along with decreased CMAP in both the median and ulnar nerves. The CMAP in lower limbs was absent. Additionally, slow SCV was observed in the median nerves, while SCV was absent in both the ulnar nerves and the tibial sensory nerve. A lumbar puncture revealed albuminocytologic dissociation in the cerebrospinal fluid (CSF), leading to a diagnosis of Guillain‐Barré Syndrome (GBS). Thereafter, the patient received two rounds of IVIG treatment and corticosteroid therapy; however, his symptoms continued to worsen. The growth and developmental history of the patient were normal, with no relevant family history. The patient had a prior history of adenotonsillar hypertrophy and sinusitis.

**FIGURE 2:**
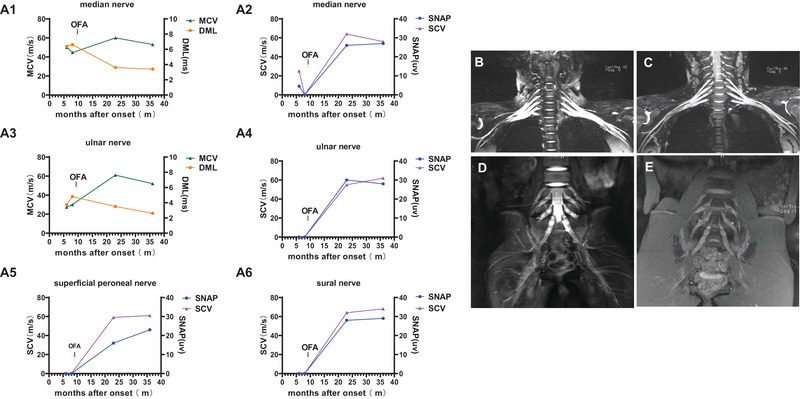
NCS changes and brachial and lumbosacral plexuses MRI images changes in case 1. (A1–A6) NCS changes in case 1 during the disease course. (A1) MCV of median nerve, (A2) SCV of median nerve, (A3) MCV of ulnar nerve, (A4) SCV of ulnar nerve, (A5) SCV of superficial peroneal nerve, (A6) SCV of sural nerve. (B and D) Lumbosacral plexuses and brachial MRI indicated nerve root thickening before ofatumumab treatment. (C and E) Nerve root thickening relieved in lumbosacral plexuses and brachial MRI 14 months after ofatumumab treatment. DML, distal motor latency; MCV, motor nerve conduction velocity; NCS, nerve conduction studies; OFA, ofatumumab; SCV, sensory nerve conduction velocity; SNAP, sensory nerve action potential amplitude.

Upon admission, neurological examination revealed symmetric sensory‐motor peripheral neuropathy, predominantly affecting the distal extremities, with tremors in both hands and atrophy of the interosseous and calf muscles, necessitating assistance for walking (Table 1). In the etiology screening, aside from the discovery of Hashimoto's thyroiditis, all other screenings related to nutritional metabolism, infections, genetics, systemic diseases, and autoimmune diseases were negative (including *PMP22*, porphyria, and urinalysis of light chains). CSF total protein level was recorded at 1220 mg/L, with negative oligoclonal bands (OB) and a 24‐h intrathecal IgG synthesis rate of 7.4 mg/day. Brain MRI showed an enlarged V–R interval, while MRI of the brachial and lumbosacral plexuses indicated nerve root thickening (Figure 2B,D). NCS primarily manifested as symmetric distal demyelinating neuropathy, characterized by symmetric, predominantly prolonged DML and reduced MCV, along with slightly decreased CMAP in both the median and ulnar nerves. This time, all SNAPs were absent in the upper limbs, and neither CMAPs nor SNAPs were detected in the lower limbs. Serum and CSF anti‐ganglioside antibodies, as well as anti‐oncological antibodies, were negative, whereas serum anti‐NF155 antibodies were positive (1:32).

**TABLE 1:** Detailed patient information with demographic characteristics in anti‐NF155 AN patients.

	Case 1	Case 2	Case 3	Case 4
**Sex**	Male	Male	Female	Male
**Age at onset (years)**	9	13	17	27
**Disease course (months)**	37	37	22	22
**Delayed diagnosis (months)**	8	2	12	15
**Vaccination before onset**	None, but deteriorated after covid‐19 vaccination	Covid‐19	None	None
**Clinical course**	Progressive	Progressive	Progressive	Progressive
**Medical history**	Adenoid hypertrophy, sinusitis	Knee surgery, hyperuricemia	—	—
**Autoimmune diseases**	Hashimoto's thyroiditis	—	—	—
**Clinical presentation**	Sensory motor	Sensory motor	Sensory motor	Sensory motor
**Clinical subtypes**	DADS	DADS	DADS	DADS
**Motor dysfunction**	Symmetric, distal	Symmetric, distal	Symmetric, distal	Symmetric, distal
**Sensory deficits**	Symmetric, distal	Symmetric, distal	Symmetric, distal	Symmetric, distal
**Tendon reflex**	Absent	Absent	Decreased	Absent
**Neuropathic pain**	None	None	None	Yes
**Sensory ataxia**	Yes	Yes	Yes	Yes
**Tremor**	Yes	Yes	Yes	Yes
**Cranial nerve**	None	None	None	None
**Autoimmune dysfunction**	Frequent urination	None	None	None
**CNS involvement**	Yes	Yes	None	None
**CSF‐protein (mg/L)**	1220	1473	1189	3613
**CSF‐cell counting (× 10**6/l)	2	2	0	4
**CSF‐OB**	Negative	Negative	Negative	Negative
**M‐protein**	Negative	Negative	Negative	Negative
**MR‐brachial plexus**	Thickening	Thickening	Thickening	Thickening
**MR‐lumbosacral plexus**	Thickening	Thickening, enhancement	Thickening	Thickening
**Brain MR**	Multiple abnormal signals in the white matter	White matter patchy t2 flair hyperintensity	UD	UD
**NF155 in blood**	1:32	1:100	1:100	1:1000
**NF155 in CSF**	—	1:3.2	1:1	1:32
**Pre‐treatment**	IVIG*2 cycles, CS	PE*3 times, RTX*3 times, CS	CS	Efgartigimod, CS
**Ofatumumab protocol**	Personalized	Personalized	Routine	Routine
**Treatment duration (months)**	14	25	11	6
**Follow‐up after ofatumumab (months)**	28	27	11	6
**Treatment at last follow‐up**	None	None	Ofatumumab	Ofatumumab

Abbreviations: AN, autoimmune nodopathy; CNS, central nerve system; COVID‐19, Coronavirus Disease 2019; CS, corticosteroid; CSF, cerebrospinal fluid; DADS, distal acquired demyelinating symmetric neuropathy; IVIG, intravenous immune globulin; NF155, neurofascin 155; OB, oligoclonal band; PE, plasma exchange; PMP22, peripheral myelin protein 22; RTX, rituximab; UD, undone.

Following the initiation of ofatumumab treatment, a personalized protocol was adopted. The CD19 B‐cell count of the patient was regularly monitored during treatment, and therapy was resumed when the count exceeded 10/µL. After the second dose of ofatumumab, the patient showed improvement and could walk independently, although foot drop persisted. The symptoms continued to improve with subsequent treatments (Figure 1A). By July 2023, after the seventh dose of ofatumumab, the patient was readmitted for reevaluation. A repeat lumbar puncture indicated normal protein levels (409 mg/L, range 150–450 mg/L), with both CSF and serum NF155 being negative. NCS revealed that DML of the median and ulnar nerves was gradually shortened, while MCV returned to normal ranges. The SCV and SNAP of the median and ulnar nerves were also gradually improved (Figure 2A1–A6). Follow‐up MRI of the brachial and lumbosacral plexuses demonstrated significant improvement in nerve root thickening (Figure 2C,E). The patient subsequently discontinued ofatumumab treatment and focused on rehabilitation. During a follow‐up visit 36 months into the disease course, the patient had mild restrictions in the movement of the distal fingers of the upper limbs, which did not affect fine motor skills such as holding chopsticks or buttoning clothing. The lower limbs still exhibited slight foot drop, with dorsiflexion strength of grade 2. Reexamination with NCS showed that conduction in the median and ulnar nerves of the upper limbs had returned to normal ranges, with recovered SCV in the lower limbs; however, CMAP in the lower limbs could not be evoked. The symptoms have remained stable, with no recurrence to date. Throughout the treatment period, the patient had good tolerance to ofatumumab and did not report any adverse reactions.

#### 3.1.2 Case 2

**FIGURE 3:**
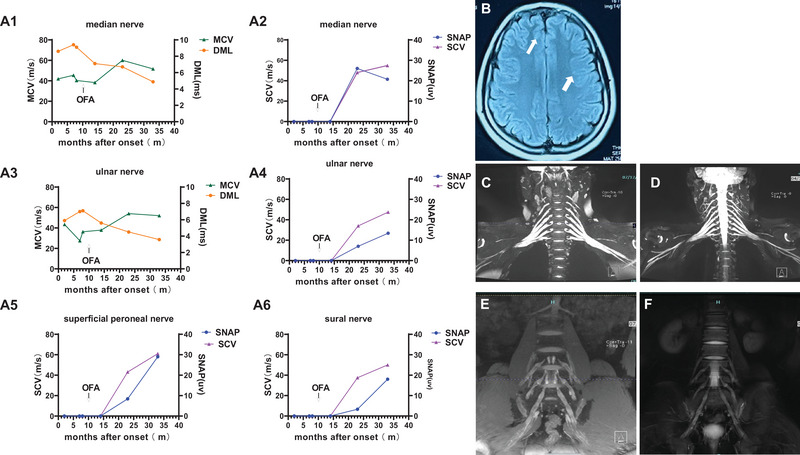
NCS changes and brachial and lumbosacral plexuses MRI images changes in case 2. (A1–A6) NCS changes in case 2 during the disease course. (A1) MCV of median nerve, (A2) SCV of median nerve, (A3) MCV of ulnar nerve, (A4) SCV of ulnar nerve, (A5) SCV of superficial peroneal nerve, (A6) SCV of sural nerve. (B) Brain MRI showed patchy high signal in the brain white matter on T2 flair. (C and E) brachial and lumbosacral plexuses MRI indicated nerve root thickening before ofatumumab treatment. (D and F) Nerve root thickening relieved in lumbosacral plexuses and brachial MRI 26 months after ofatumumab treatment. DML, distal motor latency; MCV, motor nerve conduction velocity; NCS, nerve conduction studies; OFA, ofatumumab; SCV, sensory nerve conduction velocity; SNAP, sensory nerve action potential amplitude.

A 13‐year‐old boy developed bilateral lower limb weakness 2 days after receiving the COVID‐19 vaccine in August 2021. The patient was admitted 2 months later due to progressive symptoms and inability to walk. The patient also experienced tremors in both hands, limited finger extension, and impaired fine motor skills. Episodes of dizziness were also reported. The growth and developmental history of the patient were normal, with no relevant family history. The patient had a history of knee surgery and hyperuricemia. Upon admission, physical examination revealed sensory‐motor neuropathy, mainly affecting the distal extremities. Tendon reflexes were absent, and sensory ataxia was observed. Atrophy was observed in the interosseous muscles of both hands, thenar muscles, and muscles of both calves. NCS revealed demyelinating damage in all four limbs, affecting both motor and sensory nerves, predominantly in the distal extremities (Figure 3A1–A6). Severe prolonged DML and reduced MCV were noted in both the median and ulnar nerves. All SNAPs were absent in both the upper and lower limbs, and no CMAPs were detected in the lower limbs. Brain MRI showed patchy high signal in the brain white matter on T2 flair (Figure 3B), and thickening of the nerve roots was noted in the brachial and lumbosacral plexuses (Figure 3C,E). CSF analysis indicated total protein levels of 1473 mg/L, with negative OB. In addition, the CSF IgG level was 215.0 mg/L. The NF155 antibody titer was 1:100 in the serum and 1:3.2 in CSF; however, other anti‐ganglioside antibodies were negative. No M‐protein or PMP22 abnormalities were detected, and other etiologies were ruled out, leading to a diagnosis of anti‐NF155 AN (Table 1).

Subsequently, the patient received steroids (2.5 mg administered every other day) and underwent PE thrice; however, symptoms did not improve significantly. In the fifth month of illness, the patient was treated with RTX (100 mg once a week for 3 weeks). During this treatment, the CD19^+^ B‐cell count was monitored, showing successful B‐cell depletion, while steroid therapy continued. In the tenth month of illness, as symptoms had still not improved, treatment switched to ofatumumab. The patient followed an individualized protocol based on monitoring CD19^+^ B‐cell counts. Approximately 2 months into ofatumumab treatment, clinical symptoms began to gradually improve (Figure 1B). By the 23rd month of illness (13 months into ofatumumab treatment), notable improvements in both NCS and MRI of the nerve roots were observed (Figure 3A,D,F). In addition, CSF NF155 antibodies turned negative, and CSF protein levels were assessed at 1130 mg/L. Concomitantly, the serum NF155 antibody titer decreased to 1:32. By the 35th month, serum NF155 antibodies also turned negative. Oftatumumab was then discontinued, and the patient's rehabilitation continued. Like case 1, this patient still had slight motor sequelae, including finger movement impairment and foot drop in the lower limbs, but this did not affect walking or daily life. NCS indicated good recovery of upper limb nerve function; however, CMAP in the lower motor nerves was still absent (Figure 3A). Throughout the treatment period, the patient did not report any adverse reactions to ofatumumab.

#### 3.1.3 Case 3

An 18‐year‐old female reported experiencing weakness in both lower limbs since November 2022 without seeking treatment. In June 2023, the patient sustained a right ankle sprain while walking, which exacerbated her lower limb weakness, leading to instability while walking and tremors in both hands. In October 2023, owing to worsening symptoms, the patient was admitted to the neurology department. The patient had no significant medical history, abnormalities in her growth and developmental history, or family history of related diseases. Upon admission, physical examination indicated symmetric distal peripheral neuropathy with impaired bilateral dorsiflexion, reduced deep tendon reflexes in both lower limbs, tremors in both hands, and a broad‐based gait. CSF analysis indicated increased total protein levels of 1189 mg/L, with negative OB. The CSF IgG level was 156.0 mg/L. The NF155 antibody titer was 1:100 in serum and 1:1 in CSF. NCS examination showed the absence of CMAP and SNAP in the lower limbs, a significant delay in upper limb DML, and reduced MCV and SCV. MRI of the brachial and lumbosacral plexuses indicated widespread thickening of the nerve roots. No abnormal mutations or large segment deletions were detected in the PMP22 gene. After ruling out other possible causes (infectious, genetic, metabolic, systemic autoimmune, and toxic), a diagnosis of anti‐NF155 AN was made.

Subsequently, the patient received treatment with corticosteroids and ofatumumab. Ofatumumab was administered according to the standard protocol, and the patient was monitored regularly throughout the treatment. At the 3‐month follow‐up, the symptoms of lower limb weakness had significantly improved (Figure 1C). Currently, the patient has been followed up for 11 months, and the symptoms continue to improve. NCS revealed that DML of the median and ulnar nerves was gradually shortened, while MCV returned to normal ranges. However, as in cases 1 and 2, some mild motor impairment in the distal extremities has remained. Treatment is still ongoing (Table 1).

#### 3.1.4 Case 4

A 28‐year‐old male was admitted with “numbness and weakness in both lower limbs for 15 months.” Initially, as the symptoms were mild and had little impact on his daily life, the patient did not seek medical attention. Two months before hospitalization, the patient experienced worsening symptoms, instability while walking, difficulty descending stairs, and noticed muscle atrophy in the calves. No significant past medical or family history was noted. Physical examination revealed poor muscle strength in the distal limbs, with visible atrophy in the interosseous and calf muscles. Tendon reflexes were decreased in both lower limbs, while superficial sensation in both feet was diminished, consistent with symmetric distal peripheral neuropathy. Tremors were noted in both hands, along with ataxia. NCS indicated the absence of CMAP and SNAP in the lower limbs, delayed DML, and slowed MCV in the median and ulnar nerves. SNAP in the upper limbs was absent. CSF analysis indicated a predominant increase in total protein levels at 3613 mg/L, with negative OB. The CSF IgG level was 491.0 mg/L, while the 24‐h intrathecal IgG synthesis rate was 102.25 mg/24 h. The NF155 antibody titer was 1:1000 in serum and 1:32 in CSF. MRI of the brachial and lumbosacral plexuses indicated extensive thickening of the brachial plexus, bilateral lumbosacral plexus, and sciatic nerve with increased T2 signal. No abnormal mutations or large segment deletions were identified in PMP22, and no abnormalities were detected in genome‐wide association studies. Following the exclusion of other possible causes, the patient was diagnosed with anti‐NF155 AN.

Owing to the high NF155 titer of the patient, intravenous efgartigimod treatment (10 mg/kg, once a week for 4 weeks) was recommended for eliminating pathogenic NF155 antibodies. However, the patient could not continue with efgartigimod after one treatment cycle. Consequently, a sequential treatment with ofatumumab was initiated 3 weeks after the efgartigimod cycle. Ofatumumab was administered according to the standard protocol, and the patient was regularly monitored throughout the treatment. Subsequently, the symptoms gradually improved, and the patient has been routinely followed up for 6 months (Table 1) (Figure 1D). The patient tolerated ofatumumab well during treatment, with no adverse reactions reported.

## 4 Discussion

This case series of patients with anti‐NF155 AN revealed several common characteristics between patients. The clinical features were relatively typical, primarily affecting adolescents and young adults, with all cases showing distal sensory‐motor neuropathy accompanied by tremors and ataxia. CSF protein levels were elevated above 1 g/L, and MRI findings indicated thickening of the brachial and lumbosacral nerve roots, consistent with the findings reported in the literature (Shelly et al. 2021; Menni et al. 2022). Cases 1 and 2 had abnormal white matter changes, which were considered nonspecific and presumed to be secondary to small vessel disease (Shelly et al. 2021). Despite the typical clinical features, four patients experienced diagnostic delays ranging from 2 to 15 months. Diagnostic delay is common in patients with CIDP, with a mean delay of 21.3 months (range 2–132 months), primarily due to misdiagnosis (68.3%) (Chaudhary and Rajabally 2021; Bunschoten et al. 2019). In our case series, case 1 had an acute exacerbation following vaccination and was misdiagnosed as GBS, while cases 3 and 4 were initially attributed to joint sprains. Additionally, the insidious onset and chronic progression of the disease may also be possible reasons for the diagnostic delay.

During the coronavirus disease (COVID‐19) pandemic, multiple studies have reported possible temporal associations between vaccination and the onset of GBS and CIDP (Keh et al. 2023; Baars et al. 2023). More recent studies have suggested a potential link between COVID‐19 vaccination and acute inflammatory demyelinating polyneuropathy (A‐CIDP) (Ginanneschi et al. 2023). In this study, patients 1 and 2 received inactivated COVID‐19 vaccines. Case 1 experienced disease progression, while case 2 developed symptoms after vaccination. However, it is important to note that a causal relationship has not been established.

Due to the rarity of anti‐NF155 antibody‐associated neuropathy (AN), randomized controlled trials evaluating treatment strategies are lacking. Current treatment approaches for AN predominantly follow immunomodulatory therapy for peripheral neuropathies, similar to those for CIDP. However, compared to typical CIDP patients, patients with AN show limited responsiveness to IVIG, likely due to the predominance of the IgG4 antibody isotype. IgG4 antibodies do not bind to the C1q complement component and therefore fail to activate the complement cascade. As a result, most reported cases of anti‐NF155 AN require more intensive immunotherapy, with clinical improvement typically seen only after the addition of second‐ or third‐line immunotherapy (Shelly et al. 2021). RTX, a routine B‐cell‐depletion agent, has been effectively used in anti‐NF155 AN treatment (Martin‐Aguilar et al. 2022). However, a recent study highlighted the presence of anti‐rituximab antibodies in a patient with refractory anti‐NF155 AN, resulting in a diminished response following multiple RTX infusions (Bai et al. 2023). Fully human monoclonal antibodies such as ofatumumab carry a significantly lower risk of hypersensitivity reactions compared to human‐mouse chimeric antibodies like rituximab (RTX). In our case series, no treatment‐related allergic reactions were observed. Additionally, ofatumumab offers superior clinical convenience through self‐administration at home, eliminating the need for frequent hospital‐based infusions and significantly reducing healthcare costs. In our longitudinal follow‐up, all patients successfully maintained self‐administration under outpatient monitoring.

Following treatment with ofatumumab, all patients showed clinical improvement. However, although symptomatic improvement occurred 1–2 months posttreatment in all four cases, improvements in electromyography and neuroimaging were relatively delayed. Notably, in Cases 1 and 2, despite prolonged treatment leading to clinical improvement and antibody switches to negative, as well as significant improvement in nerve hypertrophy as indicated on MRI, mild motor deficits in the distal limbs persisted, with lower limb CMAP remaining unrecordable 2 years after treatment. This indicated possible secondary axonal damage (Shelly et al. 2021), highlighting the necessity for timely diagnosis and early treatment.

Currently, no specific guidelines are available regarding the timing of medication discontinuation. A recent study found that nearly 50% of patients with anti‐NF155 AN experienced relapses within the first 4 years from symptom onset and within the first 2 years of starting treatment. The median time to relapse was 58 months (range: 11–220 months) (Shelly et al. 2021). In our study, we used an individualized approach for discontinuation. In case 1, the decision to stop medication was based on clinical symptom improvement, negative antibody tests at discontinuation, and significant improvements in CSF protein levels, imaging, and EMG results. Additionally, case 1 showed stable symptoms with no significant relapse during a follow‐up visit 1 year after treatment discontinuation, while case 2 has been currently off medication for 2 months and is still under observation.

Another important aspect of our study was the presentation of various clinical scenarios for using the monoclonal antibody ofatumumab, including as an alternative therapy after conventional immune treatments such as IVIG and corticosteroids (case 1), for refractory anti‐NF155 AN (case 2), and in sequential treatment involving neonatal Fc receptor (FcRn) inhibitors and CD20 B‐cell depletion therapy (case 3). Additionally, our research indicated that switching to the second‐generation B‐cell depleting agent, ofatumumab is an alternative for patients who do not respond well to RTX treatment. During our study, patients tolerated ofatumumab well. Throughout the treatment period, no adverse reactions, such as allergies or infections, were reported or observed.

The limitations of our study included the small number of cases and the relatively short follow‐up time for cases 3 and 4, which lacked electrophysiological and imaging data at multiple time points. As a case series involving only four patients, our study cannot determine whether OFA is more effective than RTX, and no head‐to‐head trials currently support such a comparison. An additional limitation lies in the individualized treatment protocols used in Cases 1 and 2, where delayed follow‐up testing may lead to inadequate depletion of CD20^+^ B cells, potentially compromising treatment efficacy. Well‐designed clinical trials are necessary to further evaluate the efficacy and safety of ofatumumab in treating anti‐NF155‐associated neuropathy (AN) and other AN subtypes.

## 5 Conclusion

In summary, the four patients showed clinical improvement after receiving ofatumumab treatment and did not report any adverse reactions to ofatumumab. Our research findings suggested that ofatumumab holds significant potential in treating anti‐NF155 AN and other subtypes of IgG4 antibody‐mediated AN.

## Author Contributions

**Kalam Choi**: data curation, formal analysis, validation, conceptualization, writing ‐ review and editing. **Peicai Fu**: conceptualization, writing ‐ review and editing, methodology, validation, formal analysis. **Jinyi Yan**: conceptualization, validation, formal analysis. **Zhijun Li**: writing ‐ original draft, writing ‐ review and editing, methodology.

## Ethics Statement

This study was approved by the Medical Ethics Committee of Tongji College, Huazhong University of Science and Technology ([2023]‐S171), in accordance with the ethical standards of the Declaration of Helsinki. Informed consent was obtained from all patients or their legal representatives.

## Consent

Informed consent was obtained from all patients or their legal representatives.

## Conflicts of Interest

All authors declare no conflicts of interest.

## Peer Review

The peer review history for this article is available at https://publons.com/publon/10.1002/brb3.70717.

## Data Availability

The data are available under restricted access because of data privacy laws, and access can be obtained by reasonable request to the corresponding authors.

## References

[brb370717-bib-0001] Afanasiev, V. , P. Tsouni , T. Kuntzer , et al. 2024. “Successful Autologous Hematopoietic Stem Cell Transplantation in a Refractory Anti‐Caspr1 Antibody Nodopathy.” Journal of the Peripheral Nervous System 29, no. 1: 116–119. 38123899 10.1111/jns.12610

[brb370717-bib-0002] Baars, A. E. , K. Kuitwaard , L. C. de Koning , et al. 2023. “SARS‐CoV‐2 Vaccination Safety in Guillain‐Barré Syndrome, Chronic Inflammatory Demyelinating Polyneuropathy, and Multifocal Motor Neuropathy.” Neurology 100, no. 2: e182–e191. 36127144 10.1212/WNL.0000000000201376

[brb370717-bib-0003] Bai, Y. , W. Li , C. Yan , Y. Hou , and Q. Wang . 2023. “Anti‐Rituximab Antibodies in Patients With Refractory Autoimmune Nodopathy With Anti‐Neurofascin‐155 Antibody.” Frontiers in Immunology 14: 1121705. 37056784 10.3389/fimmu.2023.1121705 PMC10086195

[brb370717-bib-0004] Broers, M. C. , L. Wieske , E. Erdag , et al. 2023. “Clinical Relevance of Distinguishing Autoimmune Nodopathies From CIDP: Longitudinal Assessment in a Large Cohort.” Journal of Neurology, Neurosurgery, and Psychiatry 95, no. 1: 52–60. 37879898 10.1136/jnnp-2023-331378

[brb370717-bib-0005] Bunschoten, C. , P. H. Blomkwist‐Markens , A. Horemans , P. A. van Doorn , and B. C. Jacobs . 2019. “Clinical Factors, Diagnostic Delay, and Residual Deficits in Chronic Inflammatory Demyelinating Polyradiculoneuropathy.” Journal of the Peripheral Nervous System 24, no. 3: 253–259. 31410938 10.1111/jns.12344

[brb370717-bib-0006] Chaudhary, U. J. , and Y. A. Rajabally . 2021. “Underdiagnosis and Diagnostic Delay in Chronic Inflammatory Demyelinating Polyneuropathy.” Journal of Neurology 268, no. 4: 1366–1373. 33170339 10.1007/s00415-020-10287-7 PMC7990867

[brb370717-bib-0007] Ginanneschi, F. , C. Vinciguerra , N. Volpi , G. Piscosquito , P. Barone , and A. Rossi . 2023. “Chronic Inflammatory Demyelinating Polyneuropathy After SARS‐CoV2 Vaccination: Update of the Literature and Patient Characterization.” Immunologic Research 71, no. 6: 833–838. 37395901 10.1007/s12026-023-09406-z

[brb370717-bib-0008] Gupta, P. , I. Mirman , S. Shahar , and D. Dubey . 2023. “Growing Spectrum of Autoimmune Nodopathies.” Current Neurology and Neuroscience Reports 23, no. 5: 201–212. 37014546 10.1007/s11910-023-01264-4

[brb370717-bib-0009] Kaegi, C. , B. Wuest , C. Crowley , and O. Boyman . 2021. “Systematic Review of Safety and Efficacy of Second‐ and Third‐Generation CD20‐Targeting Biologics in Treating Immune‐Mediated Disorders.” Frontiers in Immunology 12: 788830. 35185862 10.3389/fimmu.2021.788830 PMC8847774

[brb370717-bib-0010] Kang, C. , and H. A. Blair . 2022. “Ofatumumab: A Review in Relapsing Forms of Multiple Sclerosis.” Drugs 82, no. 1: 55–62. 34897575 10.1007/s40265-021-01650-7 PMC8748350

[brb370717-bib-0011] Keh, R. Y. S. , S. Scanlon , P. Datta‐Nemdharry , et al. 2023. “COVID‐19 Vaccination and Guillain‐Barré Syndrome: Analyses Using the National Immunoglobulin Database.” Brain 146, no. 2: 739–748. 35180300 10.1093/brain/awac067 PMC8903477

[brb370717-bib-0012] Liu, B. , J. Hu , C. Sun , et al. 2023. “Effectiveness and Safety of Rituximab in Autoimmune Nodopathy: A Single‐Center Cohort Study.” Journal of Neurology 270, no. 9: 4288–4295. 37195346 10.1007/s00415-023-11759-2

[brb370717-bib-0013] Liu, B. , L. Zhou , C. Sun , et al. 2023. “Clinical Profile of Autoimmune Nodopathy With Anti‐Neurofascin 186 Antibody.” Annals of Clinical and Translational Neurology 10, no. 6: 944–952. 37060203 10.1002/acn3.51775 PMC10270277

[brb370717-bib-0014] Martin‐Aguilar, L. , C. Lleixa , E. Pascual‐Goni , et al. 2022. “Clinical and Laboratory Features in Anti‐NF155 Autoimmune Nodopathy.” Neurol Neuroimmunol Neuroinflamm 9, no. 1: e1098. 34728497 10.1212/NXI.0000000000001098 PMC8564865

[brb370717-bib-0015] Menni, C. , A. M. Valdes , L. Polidori , et al. 2022. “Symptom Prevalence, Duration, and Risk of Hospital Admission in Individuals Infected With SARS‐CoV‐2 During Periods of Omicron and Delta Variant Dominance: A Prospective Observational Study From the ZOE COVID Study.” Lancet 399, no. 10335: 1618–1624. 35397851 10.1016/S0140-6736(22)00327-0 PMC8989396

[brb370717-bib-0016] Ren, Y. , S. Chen , and H. Yang . 2023. “Case Report: Telitacicept in Treating a Patient With NF155+ Autoimmune Nodopathy: A Successful Attempt to Manage Recurrent Elevated Sero‐Anti‐NF155 Antibodies.” Frontiers in Immunology 14: 1279808. 37965304 10.3389/fimmu.2023.1279808 PMC10642300

[brb370717-bib-0017] Scheibe, F. , L. Ostendorf , H. Pruss , et al. 2022. “Daratumumab for Treatment‐Refractory Antibody‐Mediated Diseases in Neurology.” European Journal of Neurology 29, no. 6: 1847–1854. 35098616 10.1111/ene.15266

[brb370717-bib-0018] Shelly, S. , C. J. Klein , P. J. B. Dyck , et al. 2021. “Neurofascin‐155 Immunoglobulin Subtypes: Clinicopathologic Associations and Neurologic Outcomes.” Neurology 97, no. 24: e2392–e2403. 34635556 10.1212/WNL.0000000000012932 PMC8673722

[brb370717-bib-0019] van den Bergh, P. Y. K. , P. A. van Doorn , R. D. M. Hadden , et al. 2021. “European Academy of Neurology/Peripheral Nerve Society Guideline on Diagnosis and Treatment of Chronic Inflammatory Demyelinating Polyradiculoneuropathy: Report of a Joint Task Force‐Second Revision.” European Journal of Neurology 28, no. 11: 3556–3583. 34327760 10.1111/ene.14959

[brb370717-bib-0020] Yang, M. G. , L. Xu , S. Ji , H. Gao , Q. Zhang , and B. Bu . 2022. “Tacrolimus Combined With Corticosteroids Improved the Outcome of CIDP Patients With Autoantibodies against Paranodal Proteins.” Neuropsychiatric Disease and Treatment 18: 1207–1217. 35734550 10.2147/NDT.S361461 PMC9208735

[brb370717-bib-0021] Zhou, Q. , D. Yin , J. Ma , and S. Chen . 2023. “The Therapeutic Effect of Ofatumumab in Autoimmune Encephalitis: A Case Series.” Journal of Neuroimmunology 377: 578062. 36898305 10.1016/j.jneuroim.2023.578062

